# Crowd-sourcing optimized abdomen CT protocols from 908,000 examinations in a large radiation dose registry

**DOI:** 10.1007/s00330-025-12131-w

**Published:** 2025-11-24

**Authors:** Rebecca Smith-Bindman, Taewoon Kang, Carly Stewart, Philip W. Chu, Yifei Wang, Timothy P. Szczykutowicz

**Affiliations:** 1 Department of Epidemiology and Biostatistics, University of California San Francisco, San Francisco, CA USA; 2 Department of Obstetrics, Gynecology and Reproductive Sciences, University of California San Francisco, San Francisco, CA USA; 3 Philip R. Lee Institute for Health Policy Studies, University of California San Francisco, San Francisco, CA USA; 4 Department of Radiology, University of Wisconsin, Madison, WI USA

**Keywords:** Computed tomography, Abdomen, Protocol design, Protocol optimization, Radiation dose

## Abstract

**Objectives:**

Identify routine diagnostic abdomen CT protocols in adults that achieve lower radiation doses, crowd-sourced from a large CT dose registry.

**Materials and methods:**

We retrospectively captured acquisition parameters, patient diameter, and patient size-adjusted and unadjusted radiation dose (CTDIvol and DLP). A protocol was defined as a unique combination of facility, scanner make/model, CT indication, and protocol name. We used k-means clustering to classify protocols into clusters based on similarity of average acquisition parameters (mAs, pitch, kV, collimation, scan length, phase count), each representing a distinct pattern of protocol design choices. For each cluster, we summarized the mean technical parameters and dose metrics of its constituent protocols.

**Results:**

Analyses included 907,992 exams from 1767 protocols at 132 facilities, grouped in 9 clusters. The number of protocols and exams within each cluster ranged from 62 to 508 and 15,317 to 381,457, respectively. Mean size-adjusted DLP varied threefold across clusters, ranging from 486 to 1382 mGy-cm, with no difference in patient diameter. Lowest-dosed clusters 1 and 2 minimized radiation primarily through low kV (around 100). Cluster 8 had the highest acquisition techniques (mean mAs = 338 and kV = 125), and cluster 9 had the highest dose due to phase (mean = 3.3, versus 1.1–1.6 for the remaining clusters).

**Conclusion:**

The clustering approach offers a new framework for identifying optimized protocols, while highlighting variation in practice and worst-in-class technique, e.g., using three phases for routine abdomen. These findings may guide clinicians in protocol design, by providing best-practice protocols from a large registry, as well as a method for analyzing data within their own health system.

**Key Points:**

***Question*** *Can we identify best-practice imaging protocols from a large CT registry, to offer clinicians a blueprint for optimizing radiation dose in routine abdomen CT?*

***Findings*** *Protocols were grouped into clusters using average acquisition parameters. Across clusters, patient size-adjusted radiation dose varied 486–1382 mGy-cm. Lowest-dosed protocols had one phase and low kVp.*

***Clinical relevance*** *We used crowd-sourcing and k-means clustering to identify routine abdomen CT protocols that achieve lowest radiation dose, offering a playbook for protocol design from real clinical practice. Non-indicated phases were the greatest driver of excessive radiation dose.*

**Graphical Abstract:**

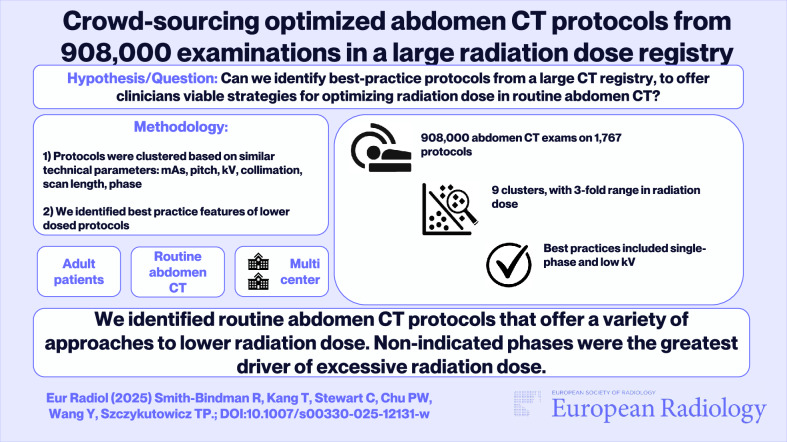

## Introduction

There is large variation in the radiation doses used for abdomen computed tomography (CT), which, as the second most common type of CT examination in Europe, contributes a higher effective dose than most other common diagnostic imaging procedures [1, 2]. A multi-national analysis of 748,846 abdomen CT examinations in adults performed for routine indications between 2015 and 2020 describes a six-fold range in the average radiation dose across 1033 unique protocols, after accounting for patient size [3]. This variation exposes patients to excess cancer risk [4, 5] and is inconsistent with a guiding principle in radiology to minimize radiation exposure while achieving appropriate image quality [6, 7].

Previous work has shown that while radiation dose varies by patient size [8], patient factors or machine characteristics only modestly drive dose variation across imaging facilities [1]. Instead, variation comes from local decisions made by clinicians as reflected in the protocols, including the selection of phases and other acquisition parameters. Well-known dose reduction approaches and technologies, such as automated kV modulation and limiting unindicated phases, are not fully utilized [1, 3, 9, 10].

While there is agreement that greater standardization of radiation doses is a clinical imperative [6, 7], there are myriad ways of selecting technical parameters to achieve sufficient image quality while minimizing radiation exposure. These choices represent trade-offs between radiation dose, image quality, scan time, and anatomical coverage, and it is not straightforward to identify the “best” protocol. Yet without a consensus-based playbook, the task of developing protocols not only consumes a large amount of time and resources for radiologists, medical physicists, and technologists, but also drives dose variation. When surveyed, individuals who oversee radiology protocols have reported feeling unable to keep up with revising protocols, and it has been estimated that maintaining updated protocols for a single indication consumes the effort of a full-time employee [11–15].

In this work, we used a crowd-sourcing approach to identify optimized protocols within a large, international radiation dose registry, and we performed cluster analyses to identify protocols that result in low radiation doses for routine abdomen CT.

## Materials and methods

Consecutive diagnostic routine abdomen CT scans performed between January 1, 2015, to March 11, 2021, in patients aged 18–99 years of age and included in the University of California San Francisco (UCSF) International CT Dose Registry (the “registry”) were included. The sample includes examinations from 132 hospitals or outpatient imaging facilities associated with 26 healthcare organizations in 7 countries. Data acquisition details have been described previously [1]. Participating sites submitted DICOM data to the registry via Radimetrics© dose management software (Bayer AG). The UCSF Institutional Review Board approved this research with a waiver of informed consent. Collaborating institutions obtained local approval or relied on UCSF approval to contribute.

These analyses focus on routine abdomen CT, including all scans of the abdomen and/or pelvis, as determined using a validated algorithm based on information in the DICOM headers, including the protocol name and study description [16]. Abdomen exams with angiography or for trauma indications were excluded, as were exams obtained for low-dose indications (e.g., suspected renal stones) or high-dose indications (e.g., liver cancer detection) [16]. A full list of indications for abdomen imaging, and the validation of the framework for assigning CT scans to categories, including routine abdomen, has been published [16].

### Data elements

Date of exam, technical parameters, and radiation dose metrics included those directly reported by the scanner (volume computed tomography dose index (CTDIvol), dose length product (DLP), kilovoltage (kV), tube current (milliamperage per second (mAs)), collimation, pitch, phase, and scan length), and those calculated in the registry (size-adjusted CTDIvol, size-adjusted DLP). Patient effective diameter is calculated and reported by Radimetrics. Scans with missing values for any of the parameters or dose metrics were eliminated (9.9% of exams). Additionally, to exclude likely erroneous outliers, we dropped all scans with values less than the 0.1th percentile or greater than the 99.9th percentile values of the technical parameters or radiation dose, and we excluded impossibly low mAs (< 5.0), impossibly low or high pitch (< 0.5 or > 3.3), and phase count > 4 (6.2% of exams). To remove scans obtained from rarely used machine models where we would have insufficient data, this study was limited to machine models with at least 5 individual scanners in the registry (10.1% of exams were excluded).

We define a protocol as a unique combination of the facility, scanner make/model, individual scanner, CT indication, and protocol name. To ensure protocols were in routine use (which we took to mean that the radiologist found image quality to be sufficient), we required a sample of at least 20 examinations per protocol for inclusion, thereby eliminating protocols that were used infrequently (potentially due to insufficient image quality for diagnosis). For each protocol, we averaged the year of study, radiation dose, and technical parameters used across the individual exams performed with that protocol because, while the protocol name might be the same, the technical parameters can vary patient to patient [17].

### Size-adjusted radiation dose

To minimize the impact of patient size on dose variation, we calculated size-adjusted CTDIvol and DLP by normalizing these metrics using the log-linear mixed regression between them and Radimetrics-reported effective diameter, with facility included as a random effect, using published methods [3]. This differs from the size-specific dose estimate (SSDE), which normalizes to obtain a consistent dose per unit of tissue as reflected in a phantom, but does not adjust dose for patient case mix across hospitals because patients with higher BMI have significantly higher SSDE than smaller patients [18, 19]. Our size normalization allows comparison of facilities by eliminating the effect of patient size on radiation dose, using the relationship between size and dose observed in our registry [3].

### Analysis

To study how variation in technical parameters drives differences in radiation dose, protocols were grouped using k-means clustering, a form of latent cluster analysis that categorizes observations into the cluster (i.e., group) with the nearest mean technical parameter value, reflected by its center point. In layman’s terms, k-means clustering groups data points that share similar features with a goal of minimizing variation within each cluster. Thus, observations within a cluster are more similar to each other than to those in other clusters. Having first summarized the protocols by average technical parameters and radiation dose metrics, we identified clusters using observed technical parameter values and sorted each protocol into the cluster closest to its own mean technical parameters [20]. Each cluster represents one pattern of behavior broadly employed in protocol design.

We performed k-means clustering analysis using six technical parameters as inputs: mAs, pitch, kV, collimation, scan length, and number of phases. These parameters were selected because they are major determinants of radiation dose and can be directly modified in protocol design. We expected these six parameters to predict the DLP and size-adjusted DLP of each cluster, reflecting the total imparted dose per examination. Since the ranges and variations of these technical parameters are quite different, their impacts on the distance to each cluster’s center point are not equally weighted. We standardized them to have a mean of 0 and a standard deviation of 1 when classifying protocols into clusters, though we show the results using their original scales.

We next determined the optimal number of clusters, ensuring it was neither too few as to obscure meaningful practice variation, nor too numerous as to introduce undue noise. We repeatedly performed k-means clustering for different numbers of clusters from 2 to 30 and calculated their total within-cluster sum of squares and cubic clustering criterion. We then plotted the sum of squares between each cluster as a function of cluster number, and identified the cluster number after which the sum of squares metric stops decreasing at an appreciable rate, identifying the elbow so that adding another cluster did not produce better modeling [20–22]. From this elbow method, we selected the optimal number, which was also a local peak of the cubic clustering criterion [23, 24].

The clusters are then numbered and sorted in tabular form by size-adjusted DLP, where cluster 1 uses the lowest radiation dose and cluster 9 the highest. To summarize the cluster, we report the mean of the technical parameters and dose metrics for its constituent protocols. While neither DLP nor CTDIvol is used in the analyses to create the clusters, they are included in the summaries to demonstrate the result of parameter choices on dose.

To graphically visualize the size, shape, and overlap of clusters as well as how combinations of parameters drive radiation dose, we defined two domains. On the *X*-axis, we have the acquisition technique domain, which encompasses mAs, pitch, and kV and is calculated as [mAs / pitch × (kV/120)^2.5^]. By this formula, a pitch > 1 or a kV < 120 will markedly reduce the X value, pushing it further to the left in the figure. On the *Y*-axis, we have the practice culture domain, which reflects phase and scan length and is calculated as [number of phases × scan length/450 mm], with 450 mm reflecting the average scan length for a single-phase routine abdomen CT. A lower number of phases or a scan length < 450 will reduce the Y value, pushing the point lower on the vertical axis. We plot each cluster along these axes as an ellipse, with a circle at the center point that is scaled in size by that cluster’s average size-adjusted DLP. The colors of the ellipses reflect their ranking in size-adjusted DLP from blue to red, reflecting the lowest to highest dose cluster, respectively. The clusters reflect the 90% prediction ellipse, illustrating a region that would contain 90% of protocols belonging to the cluster [25, 26].

We accounted for the potential for patient size to influence (confound) the variation of dose between clusters by using size-adjusted dose metrics. To assess whether the size adjustment successfully removes the influence of size, we grouped CT scans by decile based on effective diameter and then assessed the size-adjusted DLP by decile. Similarly, to explore whether the year of exam could influence (confound) the variation of dose between clusters, we assessed the proportion of exams belonging to each year, within each cluster. We additionally computed the mean size-adjusted DLP within each year, for each cluster, and assessed whether the ranking of size-adjusted DLP among clusters differed from year to year. All analyses were conducted using SAS version 9.4 and R.

## Results

A total of 907,992 routine abdomen CT examinations generated from 1767 protocols are included, with the majority (92.4%) from the United States. The average number of examinations per protocol was 514 and ranged from 20 to 21,487. Most examinations (76.9%) were obtained on a Siemens Healthineers or GE HealthCare machine. Demographic variables and descriptive statistics for the technical parameters and radiation dose metrics overall are in Tables 1 and 2.

**Table 1:** Descriptive characteristics of the routine abdomen CT examinations included in this report

		Number of CT examinations		Number of protocols	
		*N*	%	*N*	%
Total		907,992	100.0	1767	100.0
Sex					
	Men	386,963	42.6		
	Women	520,302	57.3		
	Other or unknown	727	0.1		
Age					
	18–19	11,104	1.2		
	20–29	82,975	9.1		
	30–39	109,554	12.1		
	40–49	134,006	14.8		
	50–59	169,726	18.7		
	60–69	173,364	19.1		
	70–79	134,905	14.9		
	80–89	74,489	8.2		
	90–99	17,869	2.0		
Manufacturer					
	Canon	67,135	7.4	76	4.3
	GE	405,388	44.6	890	50.4
	Philips	142,433	15.7	304	17.2
	Siemens	293,036	32.3	497	28.1
Country					
	Germany	6402	0.7	35	2.0
	Israel	17,276	1.9	15	0.8
	Japan	9469	1.0	25	1.4
	Netherlands	18,178	2.0	48	2.7
	Switzerland	1554	0.2	2	0.1
	United Kingdom	16,273	1.8	47	2.7
	United States	838,840	92.4	1595	90.3
Number of organizations		26			
Number of unique facilities		132

**Table 2:** Average technical parameters and radiation dose metrics across the included CT examinations

	Examinations		Protocols	
Included units	907,992		1767	
	Mean	Standard deviation	Mean	Standard deviation
Patient diameter (mm)	308	45	309	23
Technical parameters				
mAs	178	87	178	63
Pitch	1.0	0.3	1.0	0.3
kV	118	8	118	7
Scan length (mm)	483	85	450	82
Number of phases	1.3	0.6	1.4	0.6
Collimation	37	17	35	17
Radiation dose metrics				
Volumetric CT dose index (CTDIvol), mGy	13	7	13	5
Size-adjusted CTDIvol, mGy	11	5	12	5
Dose length product (DLP), mGy-cm	756	496	775	418
Size-adjusted DLP (mGy-cm)	657	352	669	361

The six technical parameters listed here were used as inputs in the k-means clustering analysis. *DLP* dose length product

The k-means clustering identified 9 clusters as the optimum number to characterize protocols into unique, representative groups. Summary statistics of the clusters are provided in Table 3. Each cluster encompasses between 62 to 508 unique protocols and between 15,317 to 381,457 examinations performed on those protocols. Size-adjusted DLP varies threefold across the clusters, from 486 mGy-cm (95% CI 461, 512) in cluster 1 to 1382 mGy-cm (95% CI 1260, 1504) in cluster 9.

**Table 3:** Average technical parameters for each of the 9 clusters created through k-means clustering

							Variables used as model inputs to characterize clusters						Dose Metrics			
Cluster no.	No. of protocols	Mean year of exams	No. of exams		Effective diameter mm (95% CI)		mAs	Pitch	kV	Scan length	No. of phases	Coll. (mm)	CTDIvol (mGy)	Size-Adj. CTDIvol (mGy)	DLP (mGy-cm)	Size-adjusted DLP (mGy-cm)
			N	(%)	Mean	(95% CI)	Mean	Mean	mean	Mean	Mean	Mean	Mean	Mean	Mean (95%CI)	Mean (95%CI)
1	186	2018	82,751	(9.1)	305	(295, 317)	159	0.75	105	437	1.2	36	11	10	551 (522, 580)	486 (461, 512)
2	62	2018	36,197	(4.0)	282	(262, 309)	185	1.37	102	465	1.4	35	8	8	491 (422, 561)	507 (452, 562)
3	264	2017	169,794	(18.7)	314	(299, 327)	206	1.35	120	516	1.1	33	13	10	735 (700, 771)	598 (570, 626)
4	224	2018	56,164	(6.2)	304	(284, 325)	158	0.84	120	336	1.2	27	16	15	672 (622, 722)	615 (569, 662)
5	202	2017	3562	(7.0)	309	(280, 332)	194	1.37	120	384	1.6	27	12	11	722 (676, 768)	626 (591, 661)
6	508	2018	381,457	(42.0)	310	(291, 328)	147	0.89	120	490	1.2	31	12	11	741 (713, 769)	643 (616, 670)
7	142	2018	84,950	(9.4)	306	(282, 324)	168	0.9	120	499	1.3	77	13	11	774 (722, 885)	699 (645, 753)
8	91	2017	17,800	(2.0)	338	(294, 292)	338	1.03	125	495	1.2	43	24	18	1416 (1297, 1535)	989 (916, 1062)
9	88	2017	15,317	(1.7)	303	(268, 318)	166	0.96	117	361	3.3	32	13	12	1483 (1368, 1597)	1382 (1260, 1504)
Total	1767		907,992	(100)												

The clusters are sorted by size-adjusted dose length product (DLP)

*Coll* collimation, *CTDIvol* Volumetric CT dose index

**Fig. 1:**
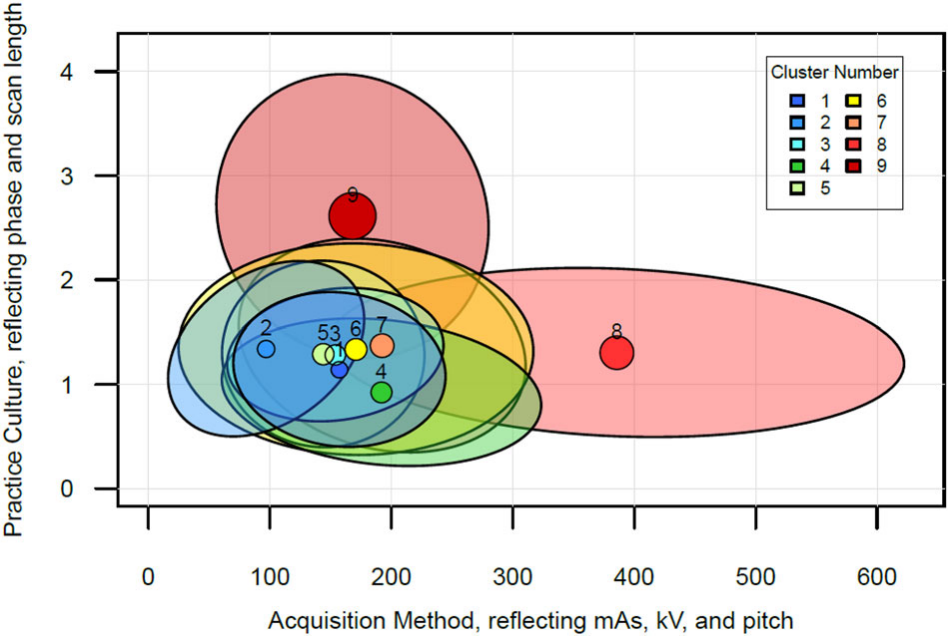
Relationship between 9 protocol-groups (clusters) identified through k-means clustering analyses using 6 technical parameters as inputs. The 90% prediction ellipse for each cluster is shown. For each of the clusters, the center bubble is proportional in size to the average size-adjusted DLP, and the clusters are colored on a gradient from blue to red, reflecting the lowest to highest size-adjusted dose length product. DLP dose length product

The relationship between the 9 identified clusters is shown in Fig. 1, and the interpretation of these configurations is aided by Table 3. Clusters 1 and 2 achieve the lowest mean size-adjusted DLP (486 and 507 mGy-cm, respectively), while clusters 8 and 9 have the highest (989 and 1382 mGy-cm, respectively) (Table 3). On the figure, these four protocols occupy different locations reflecting differences in technical parameters. Clusters 1 and 2 differ from the remaining clusters primarily by using a lower mean kV of 102–105, compared with a kV of around 120 or greater for the remaining clusters (Table 3). Cluster 1 has a slightly higher size-adjusted CTDIvol than cluster 2 (10 versus 8 mGy), but 6% lower scan length and 12% lower phase. Cluster 8 has a higher acquisition technique (resulting in a higher value on the *X*-axis), including the highest mean mAs (338) and kV (125) values amongst all clusters. Cluster 9 has about average technique in terms of mAs, kV, and pitch, but a mean phase count more than twice as high as any other cluster (3.3 phases), resulting in its position high on the *Y*-axis. Notably, the fewest number of patients are imaged with these two higher dose protocols: 2.0% of patients in cluster 8 and 1.7% in cluster 9. By comparison, 13% of examinations used protocols in clusters 1 and 2.

Protocols that fall within clusters 3–7 have average size-adjusted DLP in the range of 598–699 mGy-cm with similar technical parameter values. Cluster 6 is the largest, encompassing 508 protocols and 381,457 examinations, or 42% of all examinations. This cluster, reflecting the most common practice, results in size-adjusted DLP of 643 mGy-cm and size-adjusted CTDIvol of 11 mGy. Cluster 7 has notably high collimation (mean = 77 mm) due to a concentration of GE Revolution CT and Philips ICT 256 machines in that cluster, which perform helical scanning using 80 mm beam collimation. The use of 80 mm helical scanning is ideal for “wide axial” CT scanners for torso imaging, where high scan speeds are preferred and/or it is advantageous to increase tube output via the use of a wider collimation to minimize tube heating issues.

The concepts of acquisition technique and practice culture were meant to distinguish different approaches for dose optimization. Acquisition technique refers to practices geared at improving image quality (by decreasing visual noise), while practice culture refers to the number and length of scans. The summarized protocols in Table 3 illustrate these choices and trade-offs. Observe, for example, the differences between clusters 1 and 2. Cluster 1 has considerably lower pitch (45% less than cluster 2) but balances it with low mAs and low kV. Aside from pitch, clusters 1 and 2 have very close mAs and kV, but cluster 2 has the higher size-adjusted DLP, driven by a slightly longer scan length and 14% higher phase. Cluster 3 has interestingly both the lowest mean phase count (1.1) and the highest scan length (516 mm) of all the clusters, yet its size-adjusted dose is 18% greater than cluster 2, driven largely by a kV of 120. Cluster 4 is lowest on the *Y*-axis in Fig. 1; in Table 3, we see that this is a result of it having the shortest scan length and a low (but not the lowest) number of phases.

Ellipse size is also important. Clusters 8 and 9 have comparatively larger ellipses, reflecting greater variation within their protocols.

When compared with the mean size-adjusted DLP of the most common cluster (cluster 6), examinations in cluster 1 have a 24% reduction in dose, whereas examinations in cluster 9 have approximately 214% of the dose.

We observed no relationship between patient size and size-adjusted dose: e.g., the effective diameter in cluster 1 was 305 mm versus 303 mm in cluster 9 (Table 3). When patients were grouped by decile in effective diameter, the unadjusted dose varied considerably, as expected, while size-adjusted dose did not change with effective diameter (Table 4). For example, the size-adjusted DLP was 651 mGy-cm in the smallest decile and 649 mGy-cm in the largest. However, when patients were grouped by effective diameter and cluster, size-adjusted DLP varied markedly within each decile across clusters (Table 5). For example, patients in the first decile of size had a mean size-adjusted DLP of 473 mGy-cm in cluster 1 versus 1297 mGy-cm in cluster 9.

**Table 4:** Patient size-adjusted and unadjusted dose length product (DLP) and CT dose index volume (CTDIvol), by decile of patients’ effective diameter

Decile in effective diameter	*N*	Effective diameter (mm)		DLP (mGy-cm)				CTDIvol (mGy)			
				Unadjusted		Adjusted		Unadjusted		Adjusted	
		Mean	Median	Mean	Median	Mean	Median	Mean	Median	Mean	Median
1st	90,803	238	241	391	329	651	545	7	6	11	10
2nd	90,799	262	262	454	390	619	533	8	8	11	10
3rd	90,803	276	276	512	441	622	536	9	8	11	10
4th	90,792	288	288	577	497	634	547	10	9	11	10
5th	90,811	300	300	649	564	651	566	11	11	11	11
6th	90,791	311	311	728	640	667	586	12	12	11	11
7th	90,800	323	323	823	731	683	607	14	13	12	11
8th	90,798	337	337	940	855	696	633	16	15	12	11
9th	90,799	355	355	1097	1022	697	649	18	18	12	12
10th	90,796	393	388	1393	1282	649	601	23	22	11	11

**Table 5:** Patient size-adjusted dose length product (DLP, in mGy-cm) by decile in patients’ effective diameter and by cluster

Decile in effective diameter	Cluster number																		
	1		2		3		4		5		6		7		8		9		Total
	*N*	Mean Adj. DLP	*N*	Mean Adj. DLP	*N*	Mean Adj. DLP	*N*	Mean Adj. DLP	*N*	Mean Adj. DLP	*N*	Mean Adj. DLP	*N*	Mean Adj. DLP	*N*	Mean Adj. DLP	*N*	Mean Adj. DLP	*N*
1st	8002	473	7411	543	15,301	544	5616	671	3492	753	38,824	676	9560	774	1088	892	1509	1297	90,803
2nd	8637	466	6230	494	16,352	548	5654	634	3665	789	38,825	626	8570	738	1262	794	1604	1295	90,799
3rd	9070	471	5097	460	16,621	557	5998	664	4470	804	38,374	617	8259	747	1286	818	1628	1291	90,803
4th	8925	482	3970	453	16,924	577	6005	690	5569	819	38,451	619	8022	744	1352	841	1574	1343	90,792
5th	9064	491	3126	450	16,848	603	5814	717	6205	812	38,696	631	8001	752	1398	870	1659	1332	90,811
6th	9047	497	2587	469	17,098	633	5996	744	7056	813	37,973	642	7987	750	1419	896	1628	1337	90,791
7th	8534	506	2172	516	17,478	664	5842	765	7783	801	37,771	654	8133	738	1521	917	1566	1381	90,800
8th	8010	521	1905	598	17,192	702	5548	776	8251	777	37,901	663	8546	715	1871	935	1574	1355	90,798
9th	7440	542	1930	715	17,429	718	5282	768	8322	726	37,898	665	8720	694	2315	902	1463	1315	90,799
10th	6022	571	1769	749	18,551	648	4409	734	8749	592	36,744	621	9152	671	4288	808	1112	1151	90,796
Total	82,751		36,197		169,794		56,164		63,562		381,457		84,950		17,800		15,317		907,992

**Fig. 2:**
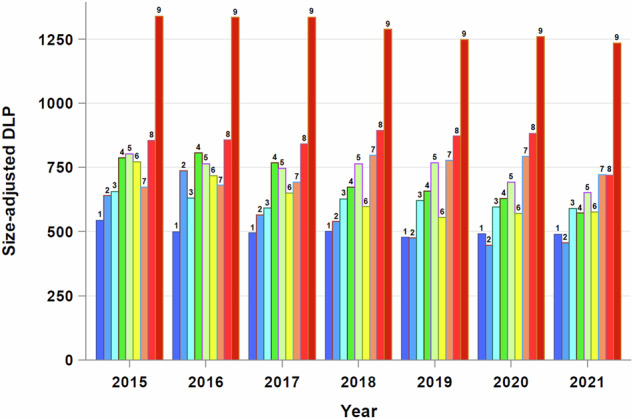
Relationship between year of exam and the 9 clusters, and mean size-adjusted dose length product (DLP, in mGy-cm) by cluster and year

Unadjusted and size-adjusted radiation dose metrics decreased approximately 20% over the study years (Supplementary Table 1). Nevertheless, within all years, exams were assigned to all clusters, demonstrating a large dose gradient across clusters within each year (Fig. 2 and Supplementary Table 2). The exceptions were cluster 2, which had more recent exams (2018–2020), and cluster 8, which had older exams (2015–2017) (Supplementary Table 2).

## Discussion

There exists a large array of approaches for performing routine abdomen CT, and these choices result in very different doses to patients. The clustering approach allowed us to identify optimally dosed protocols used across multiple sites and a large number of patients. It also demonstrated a large variation in practice, with some patients receiving excessive doses. In this sample of 1767 protocols used in nearly 1 million examinations, we observed a nearly 3-fold difference in average size-adjusted DLP between the lowest and highest protocol clusters, independent of patient size.

While patient size influences average parameter selections and was associated with a higher unadjusted dose, the lack of a relationship between patient size and size-adjusted dose indicates that patient size did not confound the dose-cluster relationship. In other words, the observed dose differences between clusters were not attributable to patient size. Furthermore, we documented that significantly lower radiation doses are feasible in larger patients, as evidenced by clusters 3 and 6. Regarding year, the distributions of exam year in clusters 2 and 8 deviated from the norm, suggesting that year may have been a confounding factor in the assignment to these clusters. However, the distribution of exam years for the other 7 clusters was relatively stable, allowing us to conclude that the primary and substantial differences in dose between clusters were not attributable to exam year. Figure 2 illustrates significant variation between clusters within each year. Even if clusters 2 and 8 were removed from the graph, the overall conclusion would remain unchanged. Cluster 2’s dose was similar to that of cluster 1, where the distribution of exam year was equal (meaning cluster 1 contained exams from all years more or less equally), while cluster 8’s dose fell midway between clusters 7 and 9, which also have equal exam year distribution.

While numerous published radiation dose benchmarks have offered median (“achievable”) and 75th percentile (“dose reference level”) doses for common indications, none of these prior works instruct how to attain the target doses [8, 27, 28]. Crowd-sourcing these clusters from a large radiation dose registry enables us to identify best practices in real, routine use and to share these with the radiology community as a playbook for radiation dose optimization. Clusters 1 through 6 in this analysis represent various viable and frequently used strategies to image routine abdomen indications in the range of published achievable doses (generally under 650 size-adjusted DLP) [8, 27]. The use of k-means clustering and graphing the resultant clusters highlights common features of the lowest dose protocols: low mAs, low kV, shorter scan length, and single phase. The features of high-dose protocols are also apparent: unnecessary phases, longer scan length, and higher than needed mAs. The takeaway message is not that every imaging provider should use the absolute lowest dose, as underlying indications and patient size may necessitate variation. Rather, providers should look to clusters 1–6 as offering a variety of avenues to image at target doses.

We did not evaluate image quality resulting from these protocols, which is a limitation of our study. However, all protocols were used in a minimum of 20 examinations (25th percentile = 38 examinations; median = 94; 75th = 350), which we presume to indicate they were acceptable to the radiologists reading the exams. Furthermore, the size-adjusted DLP for the lower-dosed clusters (particularly 3 through 6) are in the range of published achievable dose benchmarks for routine abdomen CT, suggesting these are not low-dose protocols. For example, Kanal et al give an achievable dose of 620 mGy-cm (the weighted average of abdomen and pelvis with contrast and without contrast) for patients with a diameter of 29–33 cm, approximately the median in the UCSF registry (30 cm), against which our data were size-adjusted [8]. The underlying assumption of this work is that protocols must achieve sufficient image quality for diagnosis, and that additional image quality (which comes at the cost of higher radiation dose) is generally unnecessary. Thus, assuming we have excluded truly inferior protocols, the goal is to identify those that have achieved diagnostic image quality using the lowest dose.

In addition to image quality, phase is a major driver of radiation dose. A protocol such as those in cluster 9 with relatively low acquisition technique and low CTDIvol can multiply the dose of a single-phase protocol through additional phases, with no difference in image quality. In this study, 710,803 (78.3%) of exams use 1 phase, which is appropriate for routine abdomen CT indications [10, 29].

Previous work has found kV to be the parameter most impactful on radiation dose, although it is infrequently modified from 120. Wang et al found that a 10% increase in kV led to a 29% increase in DLP, compared to an 8% or 9% DLP increase from a 10% increase in phase or mAs, respectively [9]. In clusters 1 and 2 in this analysis, a lower average kVp resulted in the lowest size-adjusted dose.

The strength of this study is its large sample of protocols and examinations from numerous, diverse institutions. There are several limitations. First, as noted, we presumed that all protocols generated sufficient image quality based on routine use of the protocols. Second, this analysis assumes all examinations were performed for routine abdomen indications, yet some may have been done for indications requiring higher or lower radiation dose, or different scan lengths. Indeed, scan length and phase count are strongly dependent on indication, and furthermore, the distribution of indications may vary by institution. However, the approach to categorizing CT examinations was previously validated and found to be 90% accurate compared to a detailed chart review, and protocols were further reviewed manually to eliminate non-routine dose indications such as those for urography, bladder cystography, any cancer assessment (including masses, lesions, nodules), acute bleeding, transplant, and others [16]. Small differences in dose and scan length between routine abdomen indications did not drive the 3-fold difference in average size-adjusted dose across the clusters. Third, as body weight is unavailable in the registry, we did not include it in our model, though body weight can affect radiation dose significantly in contrast-enhanced abdominopelvic CT. We also did not specifically address whether automatic tube current modulation or tube voltage selection techniques were used, though we presume the former was used frequently and the latter was infrequently used, as kVp rarely changed. Furthermore, it is possible that these findings are not generalizable as all contributing institutions used dose management software, perhaps reflecting heightened dose awareness. However, previous comparisons of the doses in the UCSF registry to others, such as the larger American College of Radiology Dose Index Registry, found our doses remarkably similar [8]. Finally—less a limitation than a caveat—the combination of technical parameters within the clusters in Table 3 are summaries: they are averages of the average parameters within each constituent protocol. They are not offered as recipes to follow precisely, but as guidelines for possible approaches from real practice at peer institutions.

Identifying patterns through this crowd-sourcing and clustering approach provides a useful framework for quality improvement, since dose reduction can be achieved by changes to either practice culture or acquisition technique. The outcomes highlight how variable current practice is, and how many options exist for “getting it right.” It is our hope that imaging clinicians can determine their own optimal protocols by adopting some of the practices of these lower-dose clusters: most notably, limiting phase to 1 and using kV < 120 when possible.

## Supplementary information

Electronic Supplementary Material

## Abbreviations

CT: Computed tomography
CTDIvol: Volume computed tomography dose index
DLP: Dose length product
mAs: Milliamperage per second
SSDE: Size-specific dose estimate
