# Supplementary material for: Crowd-sourcing optimized abdomen CT protocols from 908,000 examinations in a large radiation dose registry
Source: Eur Radiol. 2025 Nov 24;36(5):3454–64. doi: 10.1007/s00330-025-12131-w (PMC13086641; doi:10.1007/s00330-025-12131-w)
Supplement: Supplementary file 1 — Electronic Supplementary Material [file 330_2025_12131_MOESM1_ESM.pdf]

Crowd-sourcing optimized abdomen CT protocols from 908,000 examinations in a large radiation dose registry

ELECTRONIC SUPPLEMENTARY MATERIAL

Eur Radiol (2025) Smith-Bindman R, Kang T, Stewart C, Chu PW, Wang Y, Szczykutowicz TP.

Supplemental Table 1. Unadjusted and patient-size adjusted dose length product (DLP) over the study years, 2015-2021.

Year	Unadjusted DLP		Size-adjusted DLP	
	N	Mean (mGy)	N	Mean (mGy)
2015	110,961	825	110,961	728
2016	184,027	800	184,027	708
2017	175,782	754	175,782	662
2018	162,294	747	162,294	644
2019	147,995	714	147,995	605
2020	111,283	700	111,283	592
2021	15,650	680	15,650	581
Percent change over time:		18%		20%

Supplemental Table 2. The distribution of exam year overall and across each cluster. Column percentages are shaded from the lightest to darkest, reflecting the lowest to highest percentage, respectively.

Year	Cluster																			
	Total		1		2		3		4		5		6		7		8		9	
	N	%	N	%	N	%	N	%	N	%	N	%	N	%	N	%	N	%	N	%
**2015**	110,961	12	9,639	12	1,648	5	28,577	17	5,357	10	7,960	13	44,425	12	7,829	9	3,762	21	1,764	12
**2016**	184,027	20	14,103	17	2,540	7	36,328	21	10,629	19	15,443	24	77,044	20	18,790	22	4,641	26	4,509	29
**2017**	175,782	19	18,136	22	4,387	12	25,953	15	10,080	18	14,765	23	75,882	20	19,042	22	3,525	20	4,012	26
**2018**	162,294	18	13,682	17	8,887	25	26,788	16	10,042	18	12,097	19	68,801	18	16,936	20	2,656	15	2,405	16
**2019**	147,995	16	12,675	15	9,163	25	23,823	14	10,906	19	8,679	14	66,241	17	12,681	15	2,411	14	1,416	9
**2020**	111,283	12	12,338	15	8,130	22	25,013	15	8,071	14	3,847	6	43,817	11	8,290	10	747	4	1,030	7
**2021**	15,650	2	2,178	3	1,442	4	3,312	2	1,079	2	771	1	5,247	1	1,382	2	58	0	181	1
